# Monitoring the biological activity of abdominal aortic aneurysms *Beyond Ultrasound*

**DOI:** 10.1136/heartjnl-2015-308779

**Published:** 2016-02-15

**Authors:** Rachael O Forsythe, David E Newby, Jennifer M J Robson

**Affiliations:** British Heart Foundation Centre for Cardiovascular Science, University of Edinburgh, Edinburgh, UK

## Abstract

Abdominal aortic aneurysms (AAAs) are an important cause of morbidity and, when ruptured, are associated with >80% mortality. Current management decisions are based on assessment of aneurysm diameter by abdominal ultrasound. However, AAA growth is non-linear and rupture can occur at small diameters or may never occur in those with large AAAs. There is a need to develop better imaging biomarkers that can identify the potential risk of rupture independent of the aneurysm diameter. Key pathobiological processes of AAA progression and rupture include neovascularisation, necrotic inflammation, microcalcification and proteolytic degradation of the extracellular matrix. These processes represent key targets for emerging imaging techniques and may confer an increased risk of expansion or rupture over and above the known patient-related risk factors. Magnetic resonance imaging, using ultrasmall superparamagnetic particles of iron oxide, can identify and track hotspots of macrophage activity. Positron emission tomography, using a variety of targeted tracers, can detect areas of inflammation, angiogenesis, hypoxia and microcalcification. By going beyond the simple monitoring of diameter expansion using ultrasound, these cellular and molecular imaging techniques may have the potential to allow improved prediction of expansion or rupture and to better guide elective surgical intervention.

## Introduction

Abdominal aortic aneurysm (AAA) disease affects up to 5% of men aged between 65 and 74 years in Western Europe and may pass undetected until a patient presents with rupture. The mortality rate of ruptured AAA is over 80%,^1^ with a minority of patients surviving long enough to undergo emergency repair. The risks associated with elective AAA repair are also significant, with a 30-day mortality rate of up to 5% for elective endovascular or open repair.^2^ Decision-making at an individual patient level is therefore paramount to ensure the best clinical outcome, particularly as AAA shares risk factors for, and typically coexists with, atherosclerotic disease processes that confer substantial perioperative risk.

When planning management of a patient with an AAA, the risks of elective surgery must be weighed against the risk of rupture without intervention. Various risk prediction models have been developed to help evaluate perioperative and postoperative risk. While these scores may help evaluate risk when operative intervention is being considered, the prediction of aneurysm expansion and rupture risk in patients under surveillance remains challenging. Current knowledge of AAA progression is largely based on the tenet that rupture rate rises with increasing aneurysm diameter. However, up to one in five ruptured AAAs occur in small aneurysms (<5.5 cm),^3^ while many patients have far larger aneurysms that never rupture and a substantial proportion of patients die of non-AAA-related causes while under surveillance. We would therefore suggest that prediction of disease progression using simple measures and thresholds, such as aneurysm diameter, is too simplistic.

## Risk factors for disease occurrence and progression

Various risk factors for the development and progression of AAA disease have been identified, although it remains unclear why some patients develop AAA and others, with identical risk profiles, do not. AAA occurrence is associated with advancing age and male sex. However, female sex is also an independent predictor of accelerated disease progression. Indeed, in comparison to men, women have an increased rate of aneurysm expansion and a threefold higher risk of rupture.^4^ Moreover, when rupture takes place in women, it occurs at smaller aneurysm diameters.^5^

Cigarette smoking is the most important modifiable risk factor associated with development, expansion and rupture of AAA: more than 90% of patients with AAA are current or ex-smokers. The relative risk of aneurysm development in current smokers is at least 2,^6^ with an increased rate of expansion of the order of 15–20% in patients who continue to smoke.^7^

Although it is often said that AAA shares much of its pathophysiology with atherosclerosis, it has a number of distinct features. While traditional atherosclerotic risk factors (such as hypertension and hypercholesterolaemia) are also associated with AAA formation, diabetes mellitus is protective against its development.^8^ The reason for this remains incompletely understood but may relate to diabetes-mediated inhibition of the matrix metalloproteinases (MMPs)^9^ that are responsible for proteolytic degradation of the extracellular matrix in AAA disease. The pleiotropic effects of antidiabetic medications, including lipid homeostasis, may also be important.^10^

A strong genetic component to the development of AAA is suggested by the observation that patients with a family history of AAA have the highest risk of AAA development.^11^ Connective tissue diseases, such as Marfan's syndrome and Ehlers–Danlos syndrome, are also associated with AAA development.

## Towards improved evaluation of disease progression

Current evaluation of AAA disease progression is based on morphological risk factors for expansion—primarily, aneurysm diameter. Serial ultrasound examination is the standard method of monitoring the natural history of AAA disease, using population-derived data to predict the future expansion of an individual patient's aneurysm. The establishment of population-based ultrasound screening in the UK has been associated with a 40–50% decrease in aneurysm-related mortality.^1^ Overall, the rate of expansion and risk of rupture generally rises with increasing AAA diameter, and current guidelines recommend that elective repair should be considered when the AAA exceeds 5.5 cm in maximum anteroposterior diameter, or expansion exceeds 1.0 cm/year.

Recent data have demonstrated that many aneurysms exhibit a non-linear, discontinuous staccato growth pattern, with periods of expansion and dormancy.^12^ The specific biomechanical and biological factors that lead to this variable and patient-specific AAA expansion patterns cannot be accounted for by current aneurysm diameter measurements and recent growth trends alone. Despite this, maximum anteroposterior diameter, as measured on ultrasound or computed tomography (CT), is still considered the best clinical predictor of AAA expansion and rupture.^7^

Morphological features associated with disease progression include vessel asymmetry^13^ and a short, narrow neck configuration.^14^ The role of intraluminal thrombus in disease progression remains controversial, although plausibly a large thrombus load may induce local wall hypoxia, triggering further inflammatory activity and inducing changes in local wall strength,^15^ potentially leading to rupture. However, beyond maximum diameter none of these is validated as part of standard monitoring of patients with AAA.

As a tool for aneurysm growth surveillance, ultrasound is ideally suited since it is relatively inexpensive, readily available and avoids ionising radiation. CT offers the additional anatomical information required for surgical planning and can diagnose rupture. However, neither modality offers insight into the biological status of the aneurysm that determines disease progression. Future developments in the assessment of AAA disease should go beyond simple morphological parameters with the aim of predicting more accurately the likely evolution of the aneurysm for an individual patient.

## Pathobiological activity as targets for disease monitoring: biological ‘hotspots’

**Figure 1:**
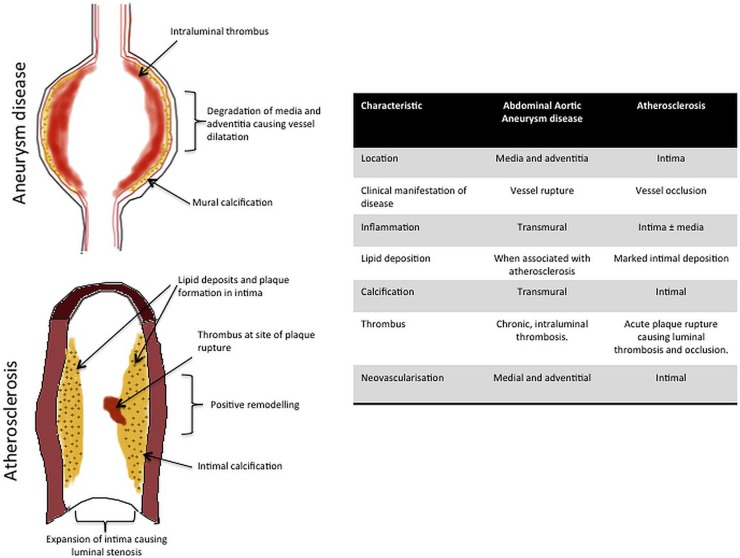
Comparison of pathobiological characteristics of atherosclerosis and abdominal aortic aneurysm (AAA) disease. While AAA disease shares similar pathobiological processes involved in atherosclerotic disease, there are notable distinctions. In particular, the location of the disease processes is an important difference, with atherosclerosis affecting primarily the vessel intima, whereas AAA disease has a predilection for the media and adventitia. The resulting clinical manifestation is that AAA disease causes vessel dilatation and rupture, whereas atherosclerosis leads to vessel stenosis occlusion. However, the common ground between these two pathological conditions means that molecular imaging techniques thus far used in the study of atherosclerotic vessels (such as the coronary and carotid arteries) may prove useful in the study of AAA disease.

Aneurysmal degeneration of the aorta involves inflammation, neovascularisation, microcalcification and proteolytic degradation of the extracellular matrix culminating in medial thinning and loss of the structural integrity of the vessel wall. Once again, there are common pathological processes involved in both AAA and atherosclerosis, but also some clear differences (figure 1). Perhaps, the most striking difference is that atherosclerosis affects the arterial intima, causing luminal narrowing, whereas aneurysmal degradation of the vessel wall predominantly affects the media and adventitia, causing vessel dilatation. Furthermore, while aneurysm disease affects only the distal abdominal aorta in 90% of cases, atherosclerosis is typically widespread involving the coronary, cerebral and peripheral circulation systems.

In studies of coronary atherosclerosis, the term ‘vulnerable plaque’ has been coined to describe plaques at risk of rupture precipitating clinical events. Such plaques are characterised by pathological features that include a thin fibrous cap, large lipid core, increased infiltration of macrophages and a reduction in smooth muscle cells.^16^ Analogous to this concept, ‘hotspots’ have been described in AAA, reflecting the focal and intense biological activity that occurs in specific regions of the aortic wall. Within these hotspots, focal neovascularisation is associated with increased inflammation and proteolysis and is present at the site of rupture.^17^ In contrast, the tensile strength of the aortic wall is not uniform and is impaired in areas of increased proteolytic activity,^18^ which varies across the aneurysm sac. These biological hotspots appear to represent areas of the aortic wall that are prone to further expansion and rupture.

A number of serum biomarkers of inflammatory and proteolytic activity have been investigated in patients with AAA including MMPs (MMP-2, MMP-8, MMP-9), interleukins (IL-6, IL-10), CRP and other potential mediators (including TNF-α and TIMP-1) that may contribute to connective tissue degradation and depletion of collagen and elastin stores. Increased levels of MMP-8 and MMP-9 have also been identified at the site of AAA rupture.^19^ However, measurement of plasma concentrations of these biomarkers has not been demonstrated to be of value in clinical risk prediction of patients and this likely reflects a lack of sensitivity. Ultimately plasma concentrations will represent activity of the entire vasculature rather than potentially small areas of focal vascular injury.

**Figure 2:**
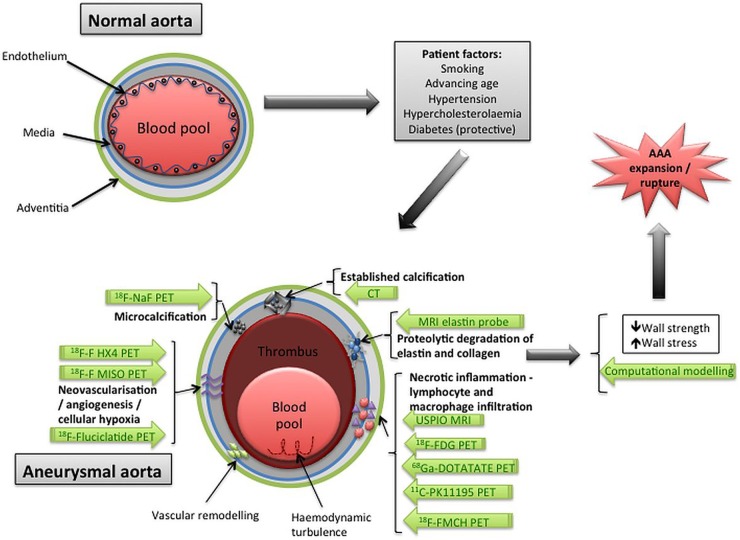
Biological targets and potential molecular imaging techniques in abdominal aortic aneurysm (AAA) disease. As well as patient factors (such as smoking, hypertension and advancing age) that contribute to the risk of AAA formation, biological processes have been identified that are implicit in aneurysm formation. The key processes are illustrated above, along with the corresponding molecular imaging techniques (written in green arrows) that have been used in experimental or clinical studies to date (primarily in the coronary arteries). The combination of patient-related risk factors and biological processes leads to increased wall stress and decreased wall strength (which can be investigated using computational modelling techniques), and all of these factors lead to aneurysm expansion and vulnerability to rupture.

The identification of biological hotspots within the aortic wall using non-invasive imaging may prove to be a more valuable approach that could enable risk prediction and, in addition to the other known risk factors for AAA progression, could form part of a patient-specific risk prediction model. However, this cannot be achieved using conventional cross-sectional imaging techniques and contrast agents. Molecular imaging techniques have the potential to provide such functional biological information and, when used in combination with structural anatomical data, may provide regional and patient-specific information on disease activity. Capitalising on our increased knowledge of the pathogenesis of cardiovascular disease, the field of ‘molecular imaging’ is one area that has expanded rapidly over the past few decades. Molecular imaging techniques apply both positron-emission tomography (PET) and magnetic resonance imaging (MRI) to identify cell-specific or process-specific probes that assess the biological function of small-scale molecular events, such as gene transcription, or identify surrogate markers for disease activity, such as inflammation and calcification. Knowledge of a relevant biological ‘target’ is a prerequisite. A suitable tracer needs to be able to bind to the target, be clinically available and offer compatibility with a clinically available imaging modality that provides appropriate spatial and tissue resolution for the disease process. Such techniques have been used in coronary artery disease and valvular heart disease to describe key processes involved in atherosclerosis including inflammation, angiogenesis and apoptosis.^20^ ^21^ These techniques can often define and identify evolving disease states at an earlier stage than conventional imaging such as the identification of vulnerable lesions associated with plaque rupture and myocardial infarction. Similarly, the identification, localisation and tracking of disease activity in patients with AAA may provide incremental risk prediction in addition to known risk factors for disease progression (figure 2).

## Macrophage activity and MRI

**Figure 3:**
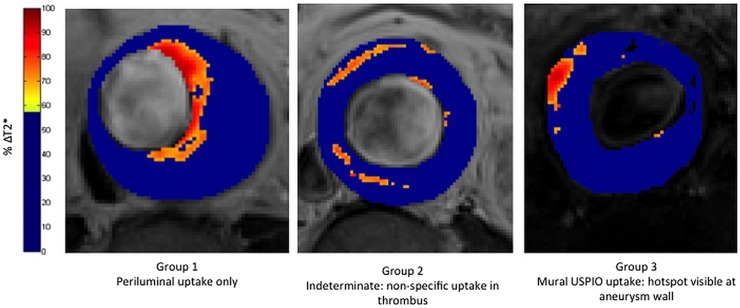
Magnetic resonance imaging (MRI) using ultrasmall superparamagnetic particles of iron oxide (USPIO) in abdominal aortic aneurysm disease. This technique is currently being investigated in the MA^3^RS study (MRI in Abdominal Aortic Aneurysms to Predict Rupture or Surgery—ISRCTN76413758).

MRI offers high spatial resolution, avoids ionising radiation and results in excellent soft tissue contrast. Molecular MRI techniques allow coregistration of molecular information with detailed anatomical information and can also be fused with CT data. Paramagnetic MRI agents (such as gadolinium) are used widely in clinical practice. When used as a blood pool agent in AAA, T2-weighted gadolinium-enhanced MRI delineates morphological features such as the blood pool, thrombus and fibrous cap^22^ whereas late enhancement reveals areas of fibrosis within vascular tissues. More recently, ultrasmall superparamagnetic particles of iron oxide (USPIOs) have been used as ‘smart’ contrast agents in cardiovascular molecular MRI studies.^23^ ^24^ USPIOs exhibit a dual benefit, in that they can be used both as an initial T1 blood-pool contrast agent as well as visualise tissue inflammation using delayed T2 and T2* imaging. Moreover, USPIOs can be safely used in patients with renal failure, whereas the use of gadolinium is contraindicated in patients with renal failure, due to the risks of nephrogenic systemic fibrosis. With a mean particle diameter of <50 nm, USPIOs are small enough to evade immediate uptake by the reticuloendothelial system and persist in the blood pool with a half-life of 10–15 h. They translocate into tissues and are slowly taken up by tissue-resident macrophages thereby accumulating at sites of inflammation. As such, they have been used to identify and track macrophage activity in situations of cardiovascular disease. In particular, they have been shown to accumulate in vulnerable and ruptured atherosclerotic plaques, but not stable plaques, and atorvastatin therapy reduces USPIO uptake and inflammation in symptomatic carotid plaques.^25^ More recently, a small pilot study of 29 patients with asymptomatic AAA (diameter between 4 and 6.6 cm) has demonstrated that focal areas of USPIO uptake in the aortic wall are associated with more rapid AAA expansion.^23^ A large, prospective multicentre trial (ISRCTN76413758) of USPIO to predict AAA rupture or surgery in patients with asymptomatic AAA (n=350) is currently in progress.^26^ This will establish whether this imaging approach has any potential clinical utility in patient monitoring (figure 3).

Further development of MRI-based techniques may include development of agents that can detect subgroups of macrophages, in addition to other aspects involved in the process of atherosclerosis and AAA disease progression.

## Macrophage activity and PET

There is a wide range of PET tracers available that can target different disease processes, many of which could be used in cardiovascular disease. PET has lower spatial resolution (∼5 mm) when compared with MRI or CT, and on its own cannot provide optimal anatomical information for detailed evaluation. However, fusion techniques (PET-CT and PET-MRI) allow the integration of molecular information obtained through PET with anatomical detail gained from CT or MRI. Macrophage activity may be tracked using cell-specific PET tracers (table 1).

**Table 1:** PET radiotracers with potential value to track disease processes in AAA

Radiotracer ^(references)^	Pathobiological process	Cellular target	Molecular target	Current clinical use	Applications and limitations
^18^F-FDG^27–33^	Inflammation	Macrophage	Glucose analogue	Yes—oncology, neurology and cardiology	Uptake influenced by local hypoxia and other resident cell types, therefore may have limited value. Widely used in cardiovascular research; has been studied in AAA disease
^68^Ga-DOTATATE^34–35^	Inflammation	Macrophage	Somatostatin receptor (subtype 2)	Experimental	No physiological uptake seen in myocardium, therefore clearly detects macrophage accumulation
^11^C-PK11195^36^	Inflammation	Macrophage	TSPO receptor	Experimental	Non-specific binding and low arterial signal density may preclude its use in clinical setting
GE180^47^	Inflammation	Macrophage	TSPO receptor	Experimental	Binding heterogeneity—10% of the population may lack binding potential due to variant in receptor
^18^F-FMCH^37–39^	Inflammation	Macrophage	Choline receptor	Yes—oncology	High uptake in liver may obscure analysis in suprarenal aorta. Lower tissue-to-background ratios than FDG, however, may identify areas of evolving inflammation distinct from areas of established calcification
^18^F-fluciclatide^47^	Angiogenesis	Endothelium, fibroblasts	Integrin α_v_β_3_	Experimental	Has been investigated in autoradiography studies of AAA disease
^18^F-FMISO^48^	Hypoxia	Macrophage	Macromolecules in hypoxic cells	Experimental	Has been used in carotid studies
^18^F-HX4^48^	Hypoxia	Macrophage	Macromolecules in hypoxic cells	Experimental	Has been used in carotid studies
^18^F-NaF^46^	Microcalcification	–	Hydroxyapatite	Yes—oncology	Overspill from nearby bone can interfere with uptake interpretation

AAA, abdominal aortic aneurysms; DOTATATE, ^68^Ga-[1,4,7,10-tetraazacyclododecane-*N*,*N*′,*N*″,*N*′-tetraacetic acid]-d-Phe^1^,Tyr^3^-octreotate; FDG, fluorodeoxyglucose; FMCH, fluoromethylcholine; FMISO, fluoromisonidazole; HX4, (3-[18F]fluoro-2-(4-((2-nitro-1H-imidazol-1-yl)methyl)-1H-1,2,3,-triazol-1-yl)-propan-1-ol); NaF, sodium fluoride; PET, positron-emission tomography; TSPO, translocator protein.

### ^18^F-Fluorodeoxyglucose

The most widely used clinical PET tracer is ^18^F-fluorodeoxyglucose (^18^F-FDG): a glucose analogue that is taken up by glucose transporters and accumulates in glucose-metabolising cells. It is used as a surrogate marker for metabolic activity and is widely used to identify tumour metastases and monitor response to cancer treatment. In addition, its value in atherosclerosis imaging (by identifying metabolically active macrophages as a marker of inflammation) has been explored largely in the coronary and carotid arteries, as it accumulates within active atherosclerotic plaques.^27^

^18^F-FDG imaging is the only clinically available tracer that has been explored in AAA disease but its value remains unclear. One small study found that AAA wall biopsies from patients with ^18^F-FDG uptake showed significantly increased inflammatory infiltrate and a reduction in the number of smooth muscle cells compared with samples from patients with no ^18^F-FDG uptake.^28^ Another small study showed that patients undergoing open repair of symptomatic AAA (n=3) had higher ^18^F-FDG uptake than asymptomatic patients (n=12), and increased ^18^F-FDG uptake correlated with histological evidence of increased inflammatory activity.^29^ Increased ^18^F-FDG uptake within the AAA was associated with more aneurysm-related clinical events compared with patients with no ^18^F-FDG uptake, and interestingly areas of ^18^F-FDG uptake correlated with increased wall stress on biomechanical modelling.^30^ The authors of these papers suggest a potential role of ^18^F-FDG PET imaging in predicting AAA expansion or rupture.

However, the value of ^18^F-FDG uptake remains controversial. Data sets are generally small and often derived retrospectively from oncology patient cohorts. Moreover, one larger study has demonstrated no difference in ^18^F-FDG uptake among patients with AAA when compared with matched controls (n=310).^31^ An inverse relationship between ^18^F-FDG uptake and future AAA expansion has been demonstrated in a small study, suggesting that aneurysms with lower metabolic activity may actually be more prone to expand.^32^

The primary drawback of ^18^F-FDG PET imaging is that the effect of local cellular hypoxia on glucose uptake may confound the PET signal that is otherwise attributed to macrophage activity,^33^ as well as the potential for spillover from adjacent structures that are metabolically active, such as muscle and liver. Thus, ^18^F-FDG PET imaging of AAA entails many potential confounding factors, lacks specificity and there is currently insufficient evidence to support its routine clinical use in predicting future AAA expansion or rupture risk.

Although other experimental PET tracers have been investigated in studies of inflammation in the context of atherosclerotic plaque, few have been investigated in AAA disease. As aneurysm disease shares some of the common pathways involved in atherosclerosis, some of these tracers may prove useful in the evaluation of AAA disease.

### ^68^Ga-DOTATATE

^68^Ga-DOTATATE (^68^Ga-[1,4,7,10-tetraazacyclododecane-*N*,*N*′,*N*″,*N*′-tetraacetic acid]-d-Phe^1^,Tyr^3^-octreotate) has a specific binding affinity for somatostatin receptors, which play a role in the modulation of inflammation and angiogenesis. Activated macrophages and damaged endothelial cells can cause overexpression of somatostatin receptors (subtype 2), and ^68^Ga-DOTATATE (which binds to these receptors) exhibits macrophage-specific binding properties that may be more valuable than ^18^F-FDG. Murine studies have demonstrated ^68^Ga-DOTATATE uptake in atherosclerotic plaques,^34^ while clinical imaging studies have demonstrated an association between ^68^Ga-DOTATATE uptake in coronary arteries and known cardiovascular risk factors such as the presence of calcified plaques and hypertension.^35^ Of note, focal uptake of ^68^Ga-DOTATATE does not always correlate with ^18^F-FDG uptake in one comparison study, suggesting that these tracers target slightly different pathobiological pathways. There have been no studies to date investigating the role of ^68^Ga-DOTATATE in AAA disease progression; however, the abundance of macrophages seen in aneurysmal aortic wall suggests that this may be a suitable tracer for evaluation.

### ^11^C-PK11195 and GE180

^11^C-PK11195 and GE180 selectively bind to the translocator protein (TSPO) and have been used primarily in the assessment of neuronal damage. However, TSPO are also highly expressed by activated macrophages. Studies have demonstrated the potential use of ^11^C-PK11195 in localising vascular inflammation in atherosclerotic disease states such as symptomatic carotid artery disease and inflamed aortic plaques.^36^ GE180 has a superior signal-to-noise ratio compared with ^11^C-PK11195; however, around 10% of people may have a genetic variant in the GE180 receptor, which leads to poor binding of the ligand. Binding heterogeneity would be a barrier to widespread use of GE180, as it would require pre-emptive genotyping. Neither tracer has been investigated in AAA disease to date.

### ^18^F-Fluoromethylcholine

^18^F-Fluoromethylcholine (^18^F-FMCH) binds to choline receptors and was introduced as a radiotracer for neuroimaging and prostate cancer. However, increased choline uptake is also seen in activated macrophages. ^18^F-FMCH uptake has been seen in murine atherosclerotic plaques, with a higher sensitivity than ^18^F-FDG,^37^ while one retrospective clinical study in oncology patients demonstrated ^18^F-FMCH uptake in structurally abnormal aortic wall,^38^ but not in areas that only exhibited established calcification. This suggests that ^18^F-FMCH may identify areas of current biological activity, distinct from areas of macroscopic calcification that are biologically inert. However, a comparison study of rat AAA has demonstrated that ^18^F-FMCH achieved lower tissue-to-background ratios than ^18^F-FDG, which was more sensitive when detecting activated macrophages.^39^

## Calcification in AAAs

Vascular calcification is associated with increased cardiovascular risk. Molecular imaging studies have suggested that inflammation and calcification occur at different stages of the atherosclerotic disease process.^40^ 18F-FDG does not always colocalise with areas of calcification seen on CT,^41^ and it is postulated that vascular calcification occurs as part of the healing response to necrotic inflammation, and is regulated in a similar manner to bone formation.^42^ Established deposits of vascular calcification are considered part of a chain of pathological processes relating to atherosclerosis.^41^ Therefore, macroscopically visible vascular calcification (macrocalcification) may correlate with overall disease burden and can be visualised on CT.

Mural calcification is common in AAA disease, is a predictor of future cardiovascular events and may also be linked with an increased risk of rupture.^43^ Calcification, resulting from necrotic inflammation, may therefore cause aneurysm wall weakness.^44^ Although widespread macrocalcification is very common in AAA disease, macrocalcification on CT does not identify those at high risk of AAA events. However, early evolving areas of calcification (microcalcification) could be a more significant and useful measurement of biological activity in the aneurysm wall, as microcalcification precedes the laying down of macrocalcification. Recent evidence suggests that plaque vulnerability is inversely associated with calcification density—established dense calcification may stabilise a plaque whereas less dense, spotty areas of microcalcification can trigger plaque rupture by causing a disproportionate increase in local stresses in the thin collagenous plaque, leading to a mismatch in compliance and vulnerability to rupture.^45^ Identifying and localising microcalcification are beyond the resolution of CT scanning and require a molecular imaging approach to detect its presence.

## Microcalcification and ^18^F-NaF PET imaging

**Figure 4:**
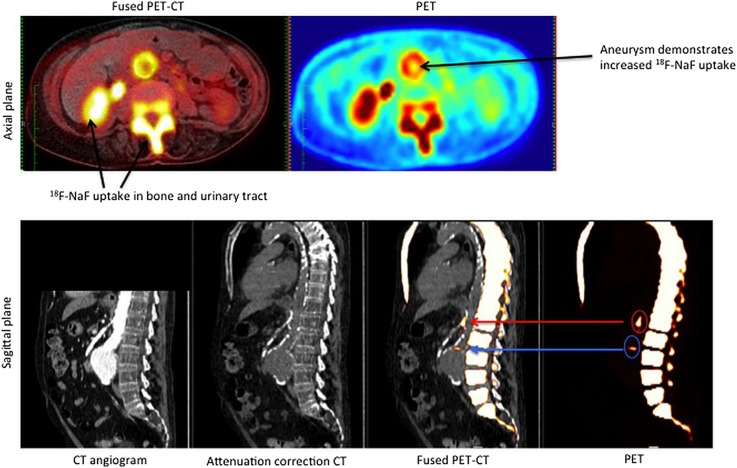
^18^F-NaF positron emission tomography–computed tomography (PET-CT) in abdominal aortic aneurysm disease. This technique is currently being investigated in the SoFIA^3^ study (Sodium Fluoride Imaging (^18^F-NaF PET-CT) in Abdominal Aortic Aneurysms—NCT02229006).

The PET radiotracer ^18^F-sodium fluoride (^18^F-NaF) has been used as a bone tracer for decades in the clinical arena. ^18^F-NaF acts as a novel marker of vascular calcification activity, by binding to hydroxyapatite, which is a key component of bone and vascular calcification. ^18^F-NaF preferentially binds to areas of developing calcification (microcalcification) and has been used to identify high-risk coronary plaques and culprit lesions in acute myocardial infarction.^46^ 18F-NaF may also be useful in patients with AAA to identify those with increased biological activity. Microcalcification represents necrotic inflammation which, in turn, weakens the vessel wall and may predispose to aneurysm expansion or rupture. By combining ^18^F-NaF PET with CT and CT angiography, identification of hotspots of increased microcalcification in the aneurysm wall could allow identification of high-risk AAAs. These data, when combined with known clinical risk factors for disease progression, may allow patient-specific evaluation of AAA expansion or rupture risk. This is currently being investigated as part of the prospective sodium fluoride imaging (^18^F-NaF PET-CT) in AAAs (SoFIA^3^) study (NCT02229006) (figure 4).

## Other targets for monitoring biological activity

Other potential PET tracers that may be useful in evaluating AAA disease do exist,^47^ although research has so far been confined largely to preclinical autoradiography studies. ^18^F-fluciclatide binds to integrins and has a high affinity for α_v_β_3_, which is expressed on endothelial cells, fibroblasts and inflammatory cells, and is known to be upregulated in angiogenesis. One autoradiography study demonstrated that ^18^F-fluciclatide detects areas of angiogenesis in AAA in vitro.^47^ 18F-FMISO (fluoromisonidazole) and ^18^F-HX4 are radiotracers that selectively identify areas of hypoxia and have been used to localise high-risk carotid artery plaque reflecting increased macrophage activity.^48^ Proteolytic degradation of collagen and elastin in the extracellular matrix is another key component in AAA formation. Gadolinium-based contrast agents, using MRI tropoelastin-binding probes, have recently shown promise in imaging impaired elastinogenesis in atherosclerotic mice^49^ and in humans following myocardial infarction.^50^

## Conclusions and future challenges

Current evaluation of AAA disease (using the basic morphological parameter of maximum aneurysm diameter) facilitates only a generic, population-based estimate of future aneurysm progression. However, more sophisticated methods of risk prediction are now required to plan timely surgical intervention, to allow appropriate screening intervals during AAA surveillance and to rationalise health resources.

While aneurysm disease shares some of the common pathways of atherosclerosis, its unique and, as yet, incompletely understood pathobiology means that further AAA-specific research is required to assess the value of promising imaging techniques that have been explored in the carotid and coronary arteries. There is a pressing need to understand the evolving biology of AAAs and its contribution to future AAA-related events. Emerging molecular imaging techniques offer the potential to add this additional biological information to the established risk factors for disease progression and may facilitate individual patient risk assessment.
